# Individual and combined effects of arbuscular mycorrhizal fungi and phytohormones on the growth and physiobiochemical characteristics of tea cutting seedlings

**DOI:** 10.3389/fpls.2023.1140267

**Published:** 2023-03-28

**Authors:** Xiubing GAO, Yan LIU, Chunyan LIU, Can GUO, Yuan ZHANG, Chiyu MA, Xueyi DUAN

**Affiliations:** ^1^ Guizhou Tea Research Institute, Guizhou Province Academy of Agricultural Science, Guiyang, Guizhou, China; ^2^ College of Horticalture and Gardening, Yangtze University, Jingzhou, Hubei, China; ^3^ Guizhou Institutes of Biology, Guizhou Academy of Sciences, Guiyang, Guizhou, China

**Keywords:** *Camellia sinensis*, mycorrhiza, antioxidase, polyamines, strigolactone

## Abstract

Both arbuscular mycorrhizal fungi (AMF) and phytohormones collectively regulate plant growth and root development, but their individual and combined effects on tea [*Camellia sinensis* (L.) O. Kuntze] cutting seedings remain unclear. This study examined the individual and combined effects of two species of AMF (*Rhizophagus intraradices*, RI and *Funneliformis mosseae*, FM) and two types of palnt hormones (strigolactones, SLs; polyamines, PAs) on tea cutting seedings, by evaluating the growth and physiobiochemical characteristics of plants treated with the AMFs and/or hormones. The results showed that inoculation with either AMF individually or hormones treatment alone could significantly enhanced mycorrhizal colonization, growth target and physiobiochemical characteristics of tea cutting seedlings. Interestingly, the addition of a combination of AMFs and hormones showed superior effects, while SL and RI exhibited the most improvements to the colonization rate, plant growth, root-morphological traits, root DHA activity, photosynthesis, chlorophyll content, soluble sugar content in leaves, and the activities of antioxidant enzymes (SOD, POD, and CAT), compared to other treatment combinations (SL + FM, PA + RI, and PA + FM). Correlation analyses revealed a significantly (*p* < 0.05) positive correlation of root AMF colonization with root-related traits (e.g., DHA, root total length, surface area, and volume) and leaf-related traits (e.g., leaf area, shoot biomass, total chlorophyll, and antioxidant enzyme activities). This study demonstrated that while the apllication of individual AMF or plant hormones had a certain good effects on most growth and physiobiochemical characteristics parameters of tea cutting seedings, the additive effect was from specific combined of AMF and plant hormones. These results highlight the possibility for combined of AMF and plant hormones to improve the asexual reproduction of tea plants *via* cuttings.

## 1 Introduction

Tea [*Camellia sinensis* (L.) O. Kuntze] is a widely consumed aromatic beverage throughout the world and an important cash crop in China (Liu et al., 2021). As a perennial evergreen woody plant that generally grows in acidic soil (Singh et al., 2010), tea leaves are rich in polyphenols, amino acids, polysaccharides, flavonoids, and other natural active substances, which have multiple health benefits, including promoting immunity and the mental health of humans (Senanayake, 2013).

Though tea plant propagation can occur *via* sexual or asexual reproduction, asexual reproduction is mainly preferred as it ensures the inheritance of the mother plant’s excellent characteristics and facilitates rapid propagation (Koyuncu and Balta, 2004; Romero, 2004). Tea plants are mainly propagated using the tea-cuttings method, which has been used for over 200 years in China (Wang et al., 2022). This asexual propagation method allows for a long breeding season and maintains the high purity of the tea variety (Wang et al., 2011). However, tea cutting seedlings with limited rooting have low survival rates. Therefore, it is important to improve the survival of tea cutting seedlings and promote their growth.

Arbuscular mycorrhizal fungus (AMFs) is a type of beneficial soil microorganism that benefits the host plant by improving root growth, nutrient absorption, soil properties, and stress resistance (Huang et al., 2011; Wipf et al., 2019). Tea plants live in symbiosis with AMFs and strongly depend on the capacity of AMFs for P uptake Shao et al, 2021. The AMF resources in the rhizosphere of tea are abundant, while *Acaulospora* and *Glomus* are the dominant AMF genera, and form good symbiotic relationships with tea plants (Liu et al., 2021). A previous study revealed that inoculation with four AMFs (*Claroideoglomus etunicatum*, *Diversispora spurca*, *D. versiformis*, and mixed-AMF) could promote the growth and biomass of tea seedlings (Shao et al., 2018). Meanwhile, inoculation with *C. etunicatum* significantly improved the leaf water content and antioxidant enzyme activity of tea plants under drought stress (Liu et al., 2020). In addition, AMF inoculation promoted the flavor and quality (e.g., catechins, amino acids, and tea polyphenols) of tea under phosphorus stress conditions (Shao et al., 2018; Cao et al., 2021a).

Increasing evidence has shown that AMF spore germination, hyphal growth, and root colonization were initiated by plant hormones (Requena et al., 2007; Sun et al., 2012; Liao et al., 2018; Pei et al., 2020). Plant hormones are known to be signaling molecules that act as important regulators of plant growth and root development, and have also been shown to play crucial roles in modulating the interactions between plants and AMFs.

Strigolactones (SLs), a new class of plant hormone, are synthesized in the plant roots and play an important role in in the regulation of plant growth, root development, and the overall morphological structure of plants (Akiyama et al., 2005). SLs are also involved in enhancing plant resistance to biotic and abiotic stresses (Sedaghat et al., 2017; Min et al., 2019). Recently, it has become clear that SLs not only stimulate seed germination, but also promote hyphae branching, activate mitochondrial function, release small molecular functional proteins of AMFs (Akiyama et al., 2005; Besserer et al., 2008; Waters et al., 2017), and improve the symbiotic associations between plant roots and soil microorganisms. SLs also enhance plant resistance to abiotic stresses (Xu et al., 2018; Yao and Waters, 2020).

Similarly, polyamines (PAs) [e.g., diamine putrescine (Put), triamine spermidine (Spd), and tetraamine spermine (Spm)]) are another class of exogenous hormones that can regulate plant growth and development, and enhance stress tolerance (Wang et al., 2015). Exogenic PAs are considered to be associated with mycorrhizal development (Jiménez Bremont et al., 2014) and were shown to significantly increase mycorrhizal colonization (Wu et al., 2012), improve antioxidant enzyme activity (SOD, POD and APX), and reduce malondialdehyde (MDA) levels in *Elymus nutans* and *Elymus sibiricus* under drought stress (Liang et al., 2020). In addition, phytohormones are important regulatory signaling factors involved in the symbiosis between AMFs and plants (Hull et al., 2021). Meanwhile, the concentration and chemical structure of SLs can control many aspects of shoot and root growth due to varied recognition by the root affecting the branching of arbuscular mycorrhizal hyphae (Waters et al., 2017).

Both AMF and plant hormones play important roles in plant growth and development, and also in enhanceing plants resistance, which showed a probably to compensate for the disadvantage of current tea cutting technology, but few studies have examined the individual and combined effects of AMF and plant hormones on the growth and propagation of tea cutting seedlings. In this study, we studied two species of AMF (*R. intraradices* and *F. mosseae*) with two types of exogenous plant hormone (GR24 and Spm) to examine their effects on the propagation, root development, photosynthesis, and antioxidant pathways in tea cutting seedings (*C. sinensis* cv. Fuding-Dabaicha).

## 2 Materials and methods

### 2.1 Experimental site and conditions

The experiment was carried out from October 2017 to November 2018, at the Guiyang Tea Garden Base (106°39’24.09”E, 26°30’52.07”N, 1127.10 m) of the Guizhou Tea Research Institute, Guizhou Province Academy of Agricultural Science, Guizhou, China. Tea cuttings were grown in a greenhouse at 30/22°C (day/night) with a relative humidity of 60% and a 14/10 h (light/dark) cycle. A seedbed (8−10 m length × 1.2−1.3 m width × 20−25 cm height) was prepared in a deep furrow field, in which a width of 10−15 cm was reserved for each region separated by furrows.

### 2.2 Test materials

Fuding white tea (*C.sinensis* (L.) O. Kuntze cv. Fuding-Dabaicha), from the Guiyang Tea Germplasm Garden (106°39’15.86”E, 26°30’10.53”N, 1116.4m) at the Guizhou Tea Research Institute, Guizhou Province Academy of Agricultural Science, China, and were most widely cultivated variety of tea tree of Guizhou provience (planting rate is more than 65%) and planted ~20 years ago, were used as the experimental materials. From October to November of 2017, when the lateral buds of the mother tea tree grew to 10−15 cm, spikes were cut. The spikes were half-lignified, annual, and strong, with long internodes, and were full axillary buds. The spikes were also free of disease and insect pests. Each cutting was 3−4 cm long with a portion of the mature leaf and plump axillary bud.

Based on the morphological identification of AMF spores, the dominant strains, *Rhizophagus intraradices* and *Funneliformis mosseae*, were used as the fungal materials. Fungi were isolated from the soil of the Guiyang Germplasm Tea Garden using the wet sieving and decanting method described by Gerdemann and Nicolson (1963). The isolated AMF spores were propagated in a greenhouse with white clover (*Trifolium repens*) hosts, and grown in a sterilized sand-soil substrate (mass ratio = 1: 1) at 30/22°C (day/night) with a relative humidity of 60% and a 14/10 h (light/dark) cycle in a greenhouse. After three months of cultivation, root segments, spores, hyphae, and the substrate containing white clover colonization were collected as the inocula (the spores density of RI and FM were 19.7 ± 1.0 and 19.4 ± 1.4 spores per 1 g of soil, respectively, using the wet sieving and decanting method).

### 2.3 Experimental design

The study was a two-factor experiment. The first factor was the fungal inoculation with *R. intraradices* (+RI), *F. mosseae* (+FM), or non-AMF inoculations (-AMF). The second factor was the application of the SL analog, GR24 (+SL), spermidine (+PA), or no hormone (-HOR). In total, there were nine treatments: -AMF – HOR, -AMF + SL, -AMF + PA, +RI – HOR, +RI + SL, +RI + PA, +FM – HOR, +FM + SL and +FM + PA. Each treatment contained three replicate plots (1.2 m in width and 5 m in length).

### 2.4 Preparation and experimental execution

Tea cuttings were grown in a seedling bed of acidic soil by laying them flat on a black plastic film and treating them with 5% formaldehyde for disinfection for 24 h before the test. The physicochemical properties of the soil were: pH, 4.51; Olsen-P, 4.52 mg/kg; available K, 275.67 mg/kg; and alkali-hydrolyzed N, 31.09 mg/kg. A layer of matrix containing AMF inocula of about 2.0 kg was spread on the furrowed field and covered with a 3−5 cm of subsoil. The subsoil was collected from a barren mountain, was mildly acidic, and was also treated with 5% formaldehyde.

The tea cuttings were disinfected by immersion in 1% carbendazim for 5 min, followed by three washes in distilled water. The planting density included a 0.5−1 cm plant spacing and 3−5 cm row spacing. After planting ~45 days, the base of tea cuttings were sprayed with 5 L of SL (1 μmol/L GR24) or PA (1 mmol/L triamine spermidine) in the hormone-treatment groups. GR24 (1 μmol/L; Umehara et al., 2008) and triamine spermidine (1 mmol/L; Wu and Zou, 2009) were dissolved in about 3-5 mL of acetone and absolute ethanol, respectively, and then prepared at 2x the desired final concentrations. The control treatments (-AMF + -HOR, -AMF + SL, -AMF + PA) were sprayed with the same weight sterilization inocula, which contained the same concentration of acetone or absolute ethanol as the solvent, respectively.

### 2.5 Measurements

After ~90 days of AMF inoculation, the survival rate, germination rate, and callus formation rate of 1,000 randomly selected tea seedlings were estimated. After ~160 days of AMF inoculation, the tea roots were stained for microscopic observation, according to the method of Phillips and Hayman (1970). The mycorrhizal colonization rate was estimated in terms of the number of roots planted with fungi and the percentage of the total number of roots observed.

After ~240 days of AMF inoculation, the tea cuttings were harvest. Then the height and leaf surface area of the plants were determined, and the above-ground and below-ground biomasses were determined after 48 h of drying at 80°C. The roots were immediately scanned with an Epson Scanner (J221A, Seiko Epson Cop., Tokyo, Japan), and root pictures were analyzed using WinRHIZO Software (2007b) (Regent Instruments, Montreal, QC Canada) to identify morphological traits.

During the sampling time between 9:00−11:00 am on sunny days, three fully functional and expanded leaves were selected for each treatment (the third leaf from the top). The gas exchange parameters of the tea seedling leaves were determined using the Li-6400 portable photosynthetic instrument (LI-COR, United States) to determine the net photosynthetic rate (Pn), stomatal conductivity (Cond), intercellular CO_2_ concentration (Ci), and transpiration rate (Tr). Each measurement was repeated three times. The chlorophyll index of the second leaf from the top of tea cuttings were estimated with the Dualex Portable Plant Polyphenol Chlorophyll Meter (Dualex Scientific+)(Zhang et al., 2022).

Root dehydrogenase activity was measured using the triphenyltetrazazole chloride (TTC) colorimetry method (Chu et al., 2007). The soluble sugar content in leaves was determined using anthrone colorimetry (Li, 2006). The leaf MDA content was measured at 532 nm and 600 nm following the thiobarbituric acid method described by Sudhakar et al. (2001). Peroxidase (POD), catalase (CAT), and superoxide dismutase (SOD) activities in leaves were determined colorimetrically, as described by He et al. (2020).

### 2.6 Statistical analysis

The data (means ± SD) obtained in this study were analyzed by two-way analysis of variance (ANOVA) in SAS software (v9.1.3) (SAS Institute Inc., Cary, NC, USA). Significant differences between treatments were compared using the Duncan’s Multiple Range Tests at the *P < 0.05* level. The Pearson’s correlation coefficients between selective variables were analyzed using SAS software. Data were graphed using SigmaPlot software (v10.0) (Systat Software, Inc., Chicago, IL, USA).

## 3 Results

### 3.1 AMF-hormone combinations improved the mycorrhizal colonization of tea cutting seedings

**Figure 1:**
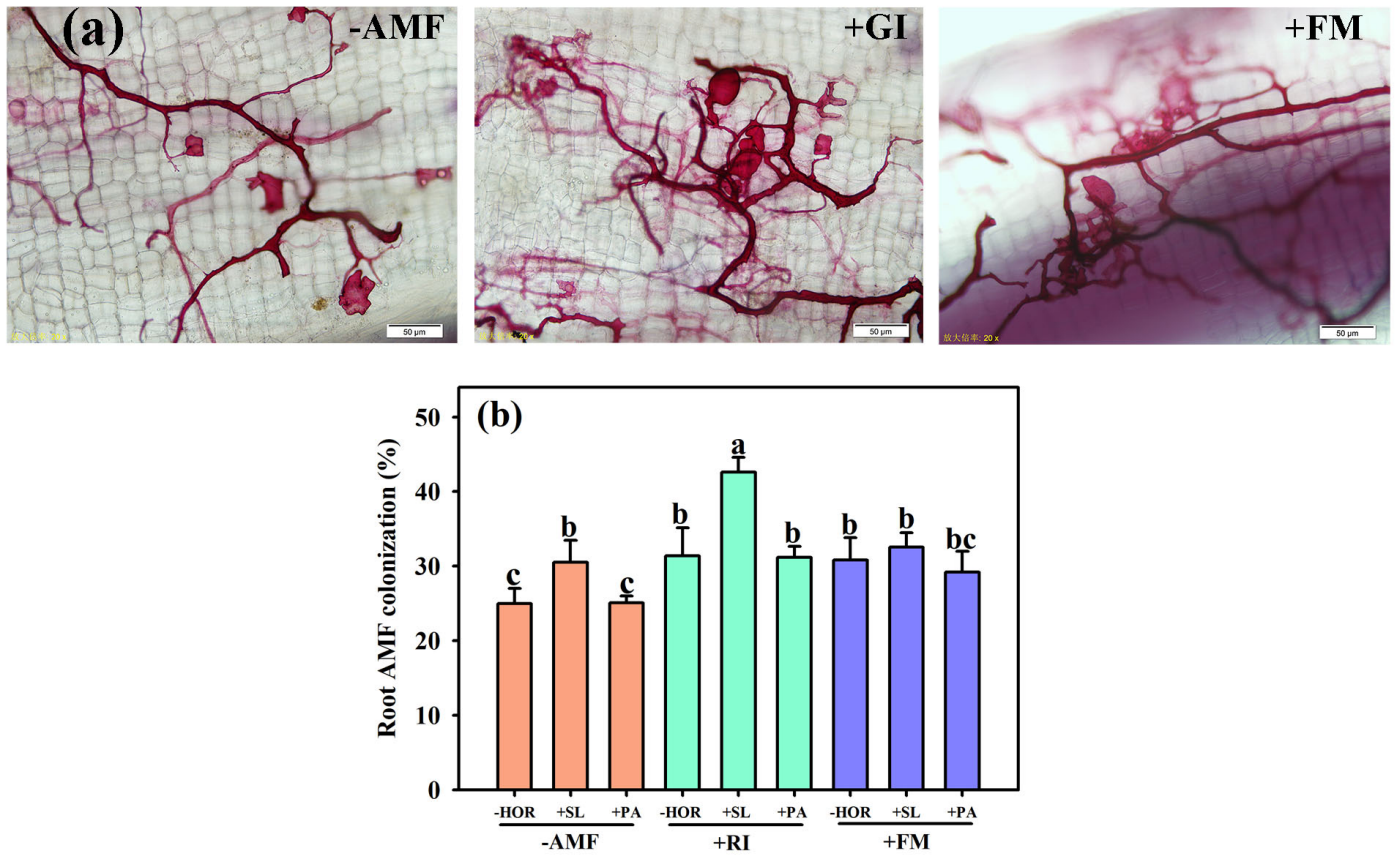
AMFs and plant hormones interaction improved the mycorrhizal colonization of tea cutting seedings. **(A)** The root system colonization structures of AMF. **(B)** Interaction effect of AMF inoculation and plant hormones on AMF colonization rates. Data bars (means ± SD, *n* = 3) indicated by different letters suggest significant differences (*P < 0.05*). AMF, arbuscular mycorrhizal fungi; RI, *Rhizophagus intraradices*; FM, *Funneliformis mosseae*; HOR, hormone; SL, strigolactones; PA, spermidine. The same treatments apply to other Figures/Tables. Different lowercase letters in a figure indicate that the mean values are significantly different (P < 0.05) from each other according to LSD test.

AMF colonization was observed in the RI-inoculated, FM-inoculated, and non-inoculated treatment groups (**Figure 1A**). Compared to -AMF – HOR and +FM + PA treatments, different treatments of RI and FM significantly improved the mycorrhizal colonization rate of tea cutting seedlings (**Figure 1B**). In addition, SL treatment increased the mycorrhizal colonization rates of non-inoculated and RI-inoculated tea cutting seedlings by 22.2% and 35.8%, respectively, but did not affect the AMF colonization rate in the FM-alone group. Irrespective of AMF inoculation, the application of PA had no significant effect on the mycorrhizal colonization rate. Two-way ANOVA was used to determine the effect of inoculated AMFs and plant hormones on the differences between variables after treatment, and a significant interaction between AMF and plant hormones was found in the mycorrhizal colonization of tea cutting seedlings (**Table 1**).

**Table 1:** Analysis of variance (ANOVA) of different variables after treatment with AMFs and hormones.

	Root colonization	Root DHA	Total chlorophyll	Leaf soluble sugar	MDA	SOD	POD	CAT
AMFs	**<0.0001**	**<0.0001**	0.1486	**<0.0001**	**0.0001**	**0.0392**	**<0.0001**	**<0.0001**
Hormones	**<0.0001**	**0.0008**	**0.0018**	0.0614	**0.0055**	**0.0003**	**0.0471**	**<0.0001**
AMFs×Hormones	**0.0363**	**0.0156**	**0.0178**	**0.0008**	0.9947	0.2869	**0.0031**	**<0.0001**

Bold values denote statistical significance at the p < 0.05 level. The “×” symbolizes the interaction of the two factors (AMFs and Hormones) within ANOVA. The bold values means there are significant or very significant differences. The same below.

### 3.2 AMF-hormone combinations improved the quality and growth indices of tea cutting seedings

Indices such as the survival rate, callus formation rate, height, leaf area, shoot biomass, and root biomass are closely associated with the quality and growth of tea cutting seedings. In this study, the survival rate of tea cutting seedings in all treatments was more than 95%, and the callus formation rate was more than 94% (**Table 2**). Compared to the -AMF - HOR treatment, the +RI + SL treatment group had the highest survival rate (98.6%) and callus formation rate (97.9%), and increased plant height of tea cutting seedings by 38.9%. None of the other treatments exhibited significant differences in plant height (*P* < 0.05). In the absense of the hormone application, RI and FM inoculations significantly increased the tea leaf surface area by 12.0% and 6.7%, respectively. Such inoculations also increased the shoot biomass and root biomass by 12.5% and 1.8%, 22.6% and 6.5% in the RI alone group and SL alone group, respectively.Without AMF inoculation, SL alone significantly increased the tea leaf surface area and shoot biomass of tea cutting seedings by 6.8% and 12.5%, respectively. PA alone significantly increased the survival rate (*P* < 0.05). Regardless of AMF inoculation, the application of PA had no significant effect on the leaf surface area. Interestingly, compared to the -AMF - HOR treatment, AMF or hormone treatments alone did not significantly affect the plant height or root biomass, while the combination of AMF and hormone treatment significantly increased the survival rate, callus formation rate, height, shoot biomass, and root biomass. In all AMF-hormone treatment groups, the best quality and growth indices of tea cutting seedings were observed in the +RI + SL treatment group. Two-way ANOVAs revealed that AMF-hormone interactions had significant effects on the leaf area and the shoot biomass of the tea cutting seedings root systems (*P* < 0.05).

**Table 2:** Effects of AMFs and hormones on the quality and growth of tea cutting seedings.

AMF treatments	Hormone treatment	Surviving rate (%)	Callus formation rate (%)	Height (cm)	Leaf area (cm^2^)	Shoot biomass (g/plant)	Root biomass (g/plant)
-AMF	-HOR	95.3 ± 0.4d	94.7 ± 1.1c	10.8 ± 2.0b	13.3 ± 0.3d	5.6 ± 0.5c	3.1 ± 0.3b
	+SL	96.1 ± 0.3cd	95.4 ± 0.5bc	10.9 ± 0.8b	14.2 ± 0.1bc	6.3 ± 0.1ab	3.2 ± 0.1ab
	+PA	96.8 ± 0.2bc	95.3 ± 0.2bc	11.0 ± 0.7b	13.6 ± 0.4cd	6.0 ± 0.2abc	3.2 ± 0.5ab
+RI	-HOR	97.6 ± 0.6ab	95.9 ± 1.0bc	12.8 ± 2.0ab	14.9 ± 0.4b	6.3 ± 0.5ab	3.4 ± 0.2ab
	+SL	98.6 ± 0.4a	97.9 ± 0.5a	15.0 ± 0.8a	16.1 ± 0.7a	6.5 ± 0.6a	3.8 ± 0.4a
	+PA	97.8 ± 1.1ab	96.6 ± 0.3abc	13.0 ± 1.5ab	14.3 ± 0.2bc	5.7 ± 0.2bc	3.0 ± 0.5b
+FM	-HOR	96.4 ± 1.1bcd	96.4 ± 1.7abc	11.3 ± 1.7b	14.2 ± 0.7bc	5.7 ± 0.2bc	3.3 ± 0.4ab
	+SL	95.2 ± 1.3d	96.7 ± 1.3ab	12.8 ± 2.7ab	14.7 ± 0.5b	5.8 ± 0.6bc	3.5 ± 0.3ab
	+PA	96.6 ± 0.8bcd	96.4 ± 1.1abc	12.6 ± 1.1ab	14.5 ± 0.3b	6.1 ± 0.5abc	3.0 ± 0.2b
Significance							
AMF		**<0.0001**	**<0.0001**	0.0058	**<0.0001**	0.2313	0.4027
Hormones		0.2318	**<0.0001**	0.2042	**0.0005**	0.1744	0.0654
AMF×Hormones		0.1784	0.1802	0.5719	**0.0470**	**0.0400**	0.5006

Data are presented as mean ± SE (n = 3). Different letters within each parameters indicate that the mean values are significantly different (P < 0.05) from each other according to LSD test. The same below.

The bold values mean that there are significant or very significant differences.

### 3.3 AMF-hormone combinations improved the root system architecture of tea cutting seedings

**Figure 2:**
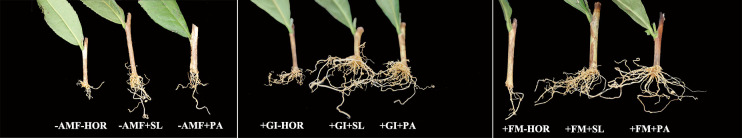
Root morphological responses of tea cutting seedings to different treatments. Compared with other treatments, +RI+SL treatment significantly improved the root-system configuration.

**Figure 3:**
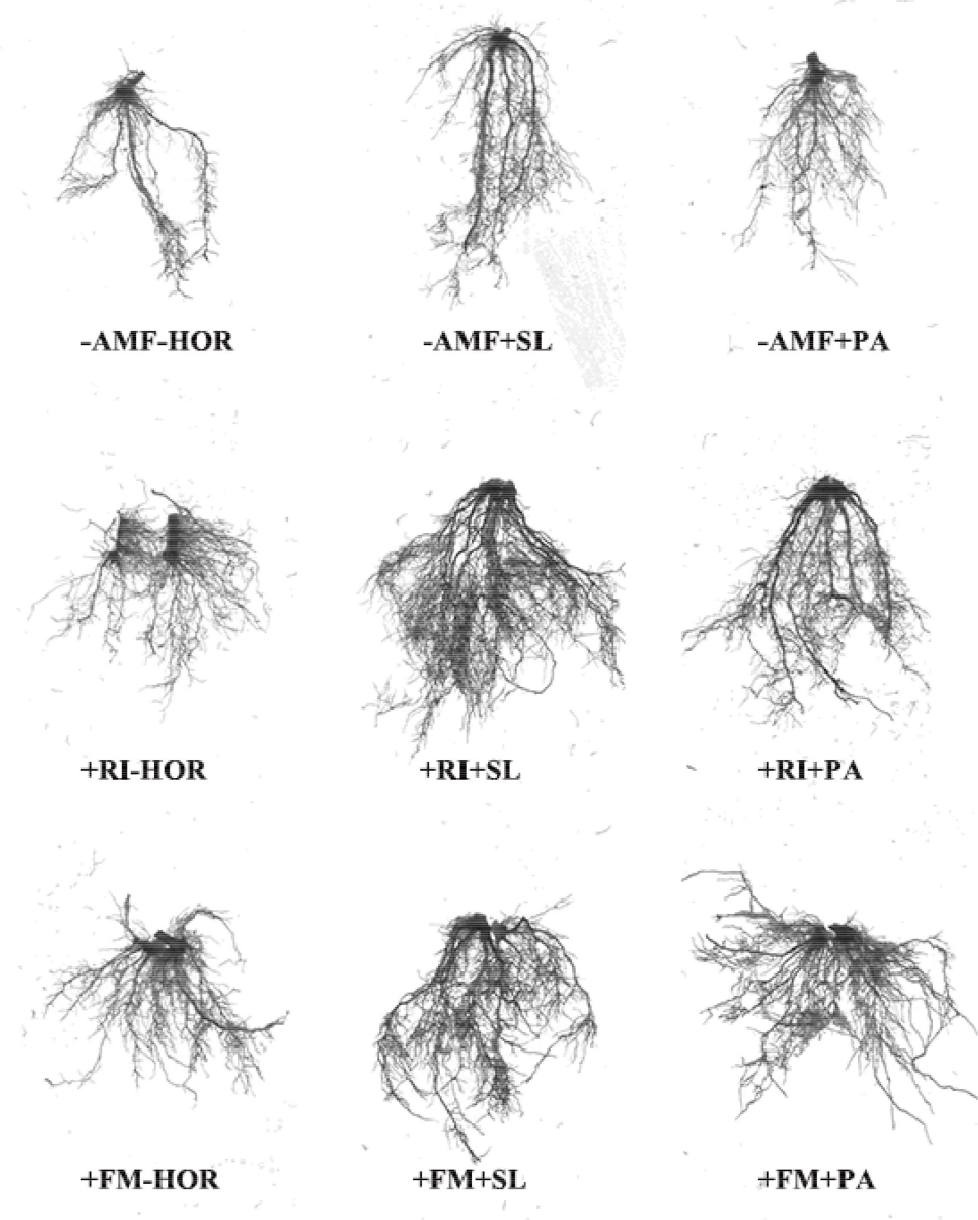
Root system architecture responses of tea cutting seedings to different treatments.

AMF and hormone interactions had different effects on the root architecture of tea cutting seedings (**Figure 2**, **3**). Compared to the -AMF - HOR treatment, RI or FM alone significantly increased the projected root area (RI, 17.2%; FM, 20.2%), average root diameter (RI, 27.3%; FM, 29.5%), and root volume (RI, 53.2%; FM, 47.7%) (**Table 3**), but had no significant impact on the total length and root surface area. Without the AMF inoculation, SL only significantly increased the average root diameter by 27.2%, while PA significantly increased the projected area and average root diameter by 17.2% and 18.2%, respectively. In combination with RI inoculation, SL significantly increased the total length, average root diameter, and root volume of tea cuttings by 61.9%, 39.3%, and 42.5%, respectively. However, the application of PA in combination with RI inoculation had no significant effect on root-system configuration. Following FM inoculation, SL significantly improved the average root diameter and root volume by 19.3% and 28.0%, respectively, while the application of PA only increased the average root diameter by 15.8%. Overall, the effect of the combination of AMF and hormones on the root system architecture was not always better than AMF inoculation or hormone treatment alone; only the specified combination of +RI + SL significantly improved root system architecture in this study (*P* < 0.05). Two-way ANOVA showed that the AMF-hormone interaction had significant effects on the total length, average diameter, and volume of the root systems of tea cutting seedings (*P < 0.05*).

**Table 3:** Interaction effect of AMFs and hormones on the root system architecture of tea cutting seedings.

AMF treatments	Hormone treatment	Total length (cm)	Projected area (cm^2^)	Surface area (cm^2^)	Average diameter (mm)	Volume (cm^3^)
-AMF	-HOR	135.8 ± 10.9c	9.9 ± 0.9c	12.3 ± 1.0b	0.44 ± 0.04d	1.09 ± 0.09d
	+SL	152.4 ± 15.0c	11.1 ± 1.2bc	13.1 ± 0.1ab	0.54 ± 0.01c	1.21 ± 0.03d
	+PA	158.0 ± 22.5bc	11.6 ± 0.7ab	13.4 ± 1.3ab	0.52 ± 0.04c	1.23 ± 0.15d
+RI	-HOR	149.7 ± 8.1c	11.6 ± 0.4ab	13.0 ± 0.6ab	0.56 ± 0.04c	1.67 ± 0.05c
	+SL	242.3 ± 31.7a	13.0 ± 0.9a	14.9 ± 1.4a	0.78 ± 0.05a	2.38 ± 0.16a
	+PA	154.0 ± 16.8bc	11.7 ± 1.1ab	13.1 ± 1.8ab	0.55 ± 0.03c	1.77 ± 0.12c
+FM	-HOR	167.9 ± 16.1bc	11.9 ± 0.7ab	12.5 ± 0.7b	0.57 ± 0.05c	1.61 ± 0.15c
	+SL	189.3 ± 22.0b	12.0 ± 0.4ab	13.4 ± 1.0ab	0.68 ± 0.06b	2.06 ± 0.20b
	+PA	145.1 ± 18.3c	11.1 ± 1.0bc	12.7 ± 0.4b	0.66 ± 0.02b	1.73 ± 0.16c
Significance						
AMF		**0.0061**	**0.0293**	0.2537	**<0.0001**	**<0.0001**
Hormones		**0.0001**	0.0827	0.0752	**<0.0001**	**<0.0001**
AMF×Hormones		**0.0025**	0.1306	0.5409	**0.0013**	**0.0062**

Data are presented as the mean ± SE (n = 3).

The bold values mean that there are significant or very significant differences.

### 3.4 AMF-hormone combinations increased root DHA activity

**Figure 4:**
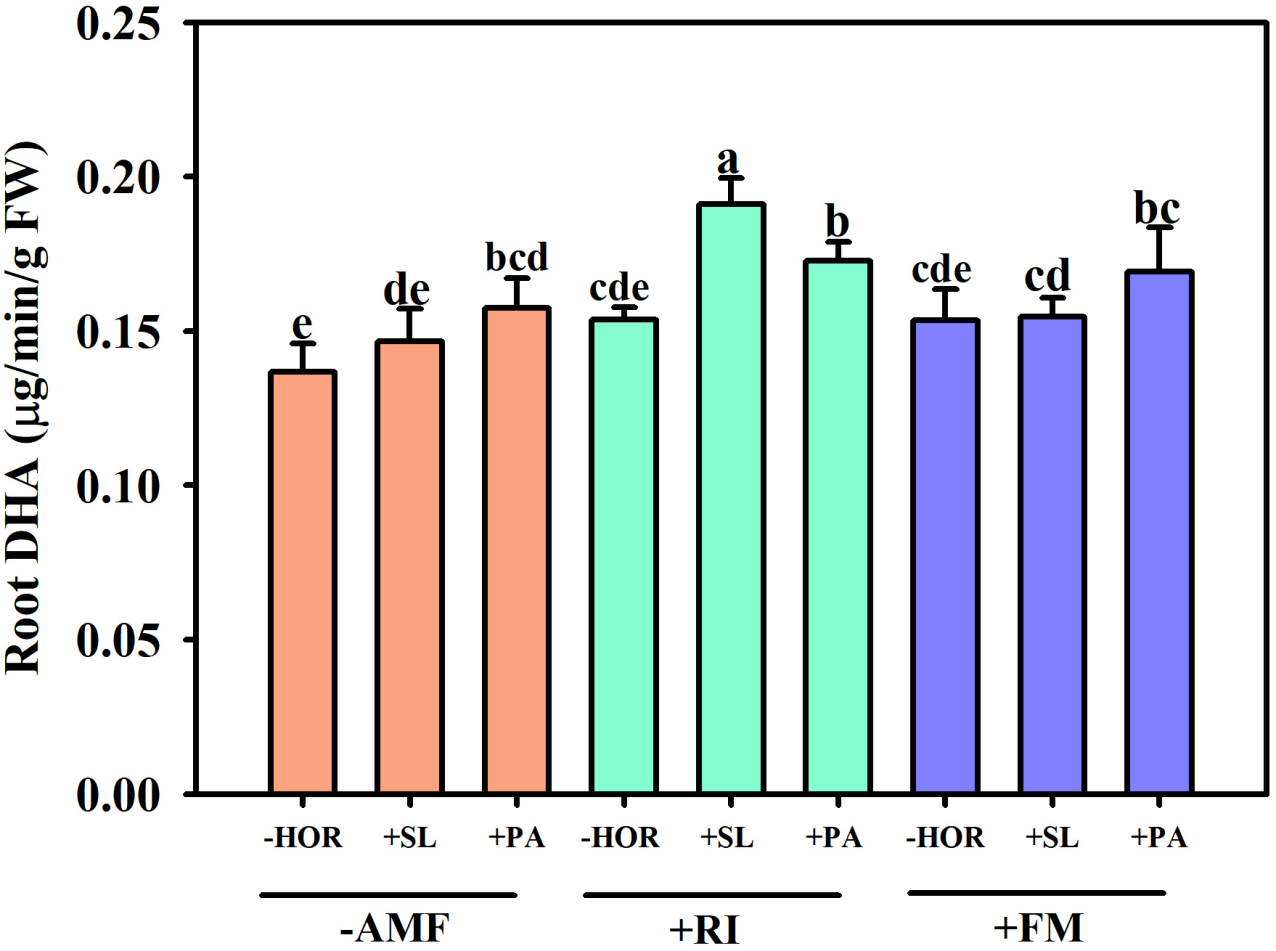
AMFs and plant hormones interaction significantly increased the root DHA activity. Different lowercase letters in a figure indicate that the mean values are significantly different (P < 0.05) from each other according to LSD test. The same below.

In the absence of plant hormone treatment, inoculation with RI or FM had no significant effect on root DHA activity (**Figure 4**). However, following inoculation with AMF, PA significantly increased root DHA activity by 15.1% (*P* < 0.05), while SL had no significant effect compared to the corresponding control (-HOR + -AMF). Compared to the other combined treatments, the +RI + SL group exhibited the highest increase in root DHA activity at 24.3% compared to the corresponding control (-HOR + RI). The +RI + PA and +FM + PA treatment groups also had significantly higher root DHA activities than the controls (*P* < 0.05). This turned out to be the case as the specified combination of AMFs and hormones had a significant effect on the root DHA activity.

### 3.5 AMF-hormone combinations affected the photosynthesis of tea cutting seedings

As shown in **Table 4**, in the absence of hormone treamtents, RI inoculation significantly improved the net photosynthetic rate and stomatal conductance of tea cutting seedlings by 36.2% and 47.3%, respectively. Additionally, FM inoculation significantly improved the Pn, Cond, and Tr by 32.6%, 39.6%, and 41.3%, respectively. In the absence of AMF inoculation, SL had no significant effect on photosynthetic parameters, while PA only significantly increased Cond (30.8%). Treatement with +RI + SL significantly improved the Pn, Cond, and Tr by 43.8%, 58.2%, and 89.9%, rewpectively, while +RI + PA significantly improved Pn, Cond, and Tr by 37.5%, 29.9%, and 51.9%, respectively. Treatment with +FM + SL increased Pn by 21.2%, while treatment with +FM + PA significantly improved the Cond and the transpiration rate by 27.6% and 26.5%, respectively. No significant difference in Ci was observed in any treatment. Two-way ANOVA revealed that AMF-hormone interactions had significant effects on stomatal conductance and the transpiration rate of tea cutting seedings (*P* < 0.05).

**Table 4:** Effects of AMFs and hormones on photosynthesis in tea cutting seedings.

AMF treatments	Hormone treatments	Pn(µmol/m^2^/s)	Cond (mmol/m^2^/s)	Ci (μmol/mol)	Tr (mmol/m^2^/s)
-AMF	-HOR	4.70 ± 1.40e	0.091 ± 0.035f	317.3 ± 21.5a	1.04 ± 0.33f
	+SL	5.59 ± 0.74de	0.101 ± 0.026ef	309.4 ± 19.3a	1.13 ± 0.37f
	+PA	5.64 ± 0.87de	0.119 ± 0.015de	322.7 ± 9.0a	1.20 ± 0.18ef
+RI	-HOR	6.40 ± 0.74cd	0.134 ± 0.016d	313.3 ± 26.0a	1.29 ± 0.25ef
	+SL	9.20 ± 0.81a	0.212 ± 0.051a	316.3 ± 17.1a	2.45 ± 0.43a
	+PA	8.80 ± 1.22a	0.174 ± 0.031b	311.1 ± 16.2a	1.96 ± 0.45b
+FM	-HOR	6.23 ± 1.57cd	0.127 ± 0.019d	314.2 ± 14.0a	1.47 ± 0.46de
	+SL	7.55 ± 1.49b	0.140 ± 0.017cd	311.4 ± 8.1a	1.61 ± 0.33cd
	+PA	7.08 ± 2.20bc	0.162 ± 0.030bc	318.1 ± 14.3a	1.86 ± 0.40bc
Significance					
AMF		**<0.0001**	**<0.0001**	0.7625	**<0.0001**
Hormone		**<0.0001**	**<0.0001**	0.4883	**<0.0001**
AMF×Hormone		0.0676	**0.0001**	0.4635	**<0.0001**

Data are presented as the mean ± SE (n = 10). Pn, net photosynthetic rate; Cond, stomatal conductivity; Ci, intercellular CO_2_ concentration; Tr, transpiration rate.

The bold values mean that there are significant or very significant differences.

### 3.6 AMF-hormone combinations affected chlorophyll and soluble sugar contents in tea cutting seedings leaves

**Figure 5:**
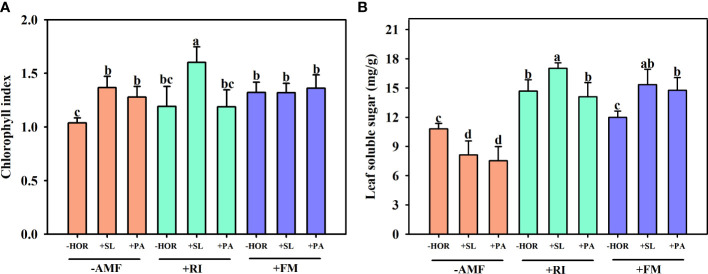
AMFs and plant hormones interaction had significant effects on chlorophyll indexes and soluble sugar contents in tea cutting seedings leaves. Effects of AMFs and hormones on **(A)** total chlorophyll index and **(B)** leaf soluble sugar content. Different lowercase letters in a figure indicate that the mean values are significantly different (P < 0.05) from each other according to LSD test.

As shown in **Figure 5A**, compared to the -AMF - HOR treatment group, RI inoculation alone had no significant effect on the chlorophyll content, while FM inoculation alone significantly increased the chlorophyll content by 28.2%. Treatment with -AMF + SL and -AMF + PA significantly increased the chlorophyll content by 31.9% and 23.4%, respectively. Treatment with +RI + SL significantly increased the chlorophyll content by 34.6%, while +RI + PA had no significant effect. Treatment with +FM + SL and +FM + PA did not affect the chlorophyll content.

Concerning the soluble sugar content in the leaves of tea cutting seedings, the application of SL and PA without AMF inoculation significantly reduced the soluble sugar contents of leaves (**Figure 5B**) by 32.8% and 43.2%, respectively. Treatment with +RI + SL significantly increased the soluble sugar content by 16.0%, while +RI+PA had no significant effect. Treatment with +FM + SL and +FM + PA significantly increased the soluble sugar content of leaves by 28.0% and 23.2%, respectively. In the absence of hormone treatment, RI inoculation significantly increased the leaf content of soluble proteins by 35.9%, while FM inoculation had no effect. In addition, AMF and hormone interactions had significant effects on the total chlorophyll and soluble sugar contents of tea cutting seedings (**Table 1**).

### 3.7 AMF-hormone combinations increased the POD, SOD, and CAT activities of tea cutting seedings

**Figure 6:**
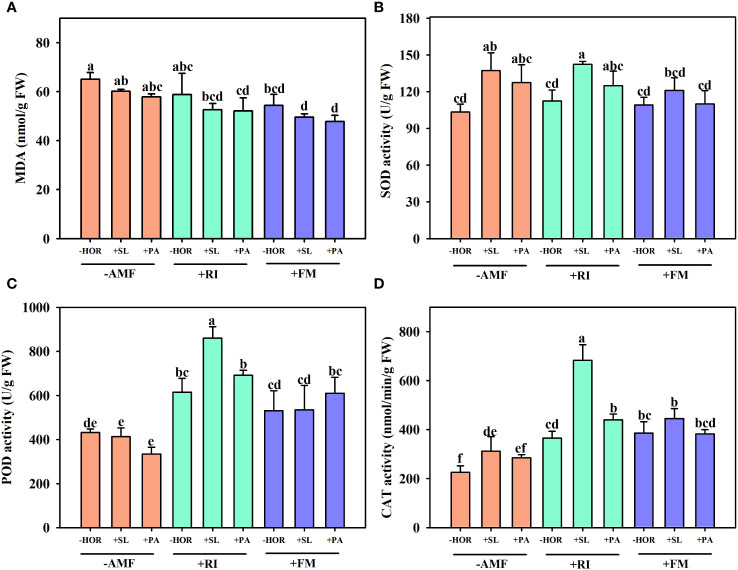
AMFs and plant hormones interaction showed the highest increase in POD, SOD, and CAT activities. Effects of AMFs and hormones on **(A)** MDA content and the leaf activities of **(B)** SOD, **(C)** POD, and **(D)** CAT. Different lowercase letters in a figure indicate that the mean values are significantly different (P < 0.05) from each other according to LSD test.

Compared to the -AMF - HOR treatment, +RI + SL or +RI + PA treatments did not change the MDA content, while FM inoculation significantly reduced MDA concentrations by 19.7% (**Figure 6A**). When inoculated with RI and FM, spraying SL or PA had no significant effect on MDA concentration.

Similar to MDA concentrations, RI inoculation and FM inoculation alone did not affect leaf SOD activities compared to the -AMF - HOR treatment group (**Figure 6B**). However, the application of SL significantly increased SOD activities by 32.8%, while PA treatment caused no change. Treatment with +RI + SL significantly increased SOD activity by 26.7%, while treatment with +RI + PA caused no change. Neither +FM + SL nor +FM + PA treatments affected SOD activities.

In the absence of hormone treatments, RI inoculation alone significantly increased POD activity by 45.2%, while FM inoculation caused no effect (**Figure 6C**). Without AMF or with FM inoculation, the application of SL or PA had no significant effect on POD activities, while treatment with +RI + SL significantly improved POD activity by 40.1%.

In the absence of hormone treatment, RI inoculation or FM inoculations significantly improved the leaf CAT activity by 62.2% and 71.1%, respectively (**Figure 6D**). Treatment with -AMF + SL significantly improved leaf CAT activity by 38.2%, while -AMF + PA had no effect. When combined with RI inoculation, both SL and PA treatment significantly improved the leaf CAT activity by 87.1% and 20.3%, respectively. When combined with FM inoculation, SL or PA treatment exhibited no significant changes in leaf CAT activity.

Compared to the other treatments, +RI + SL treatment caused the highest increase in POD, SOD, and CAT activities. Thus, AMF-hormone interactions significantly (*P* < 0.01) affected the leaf POD and CAT activities of tea cutting seedings (**Table 1**).

### 3.8 Correlation analysis

**Figure 7:**
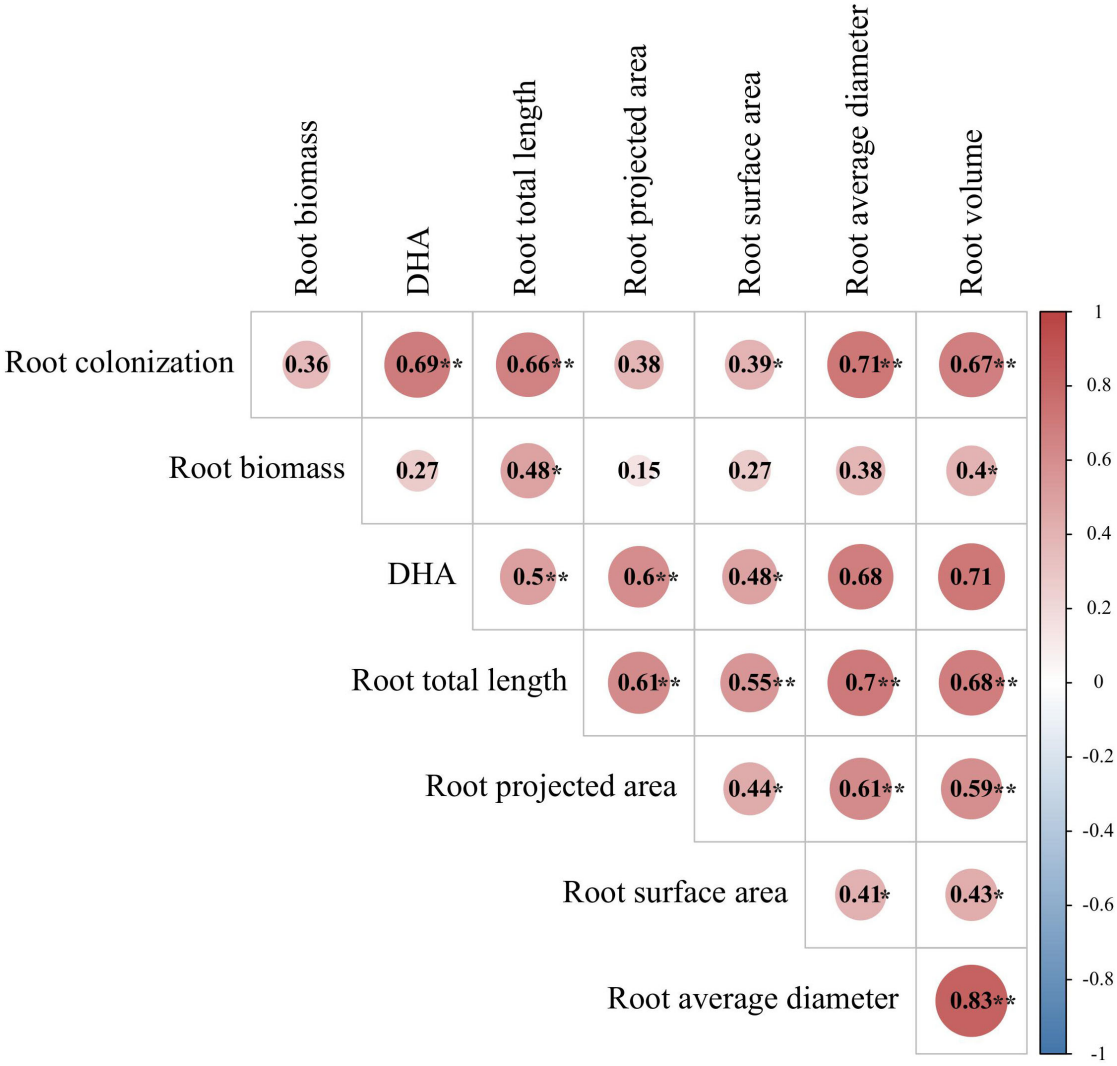
Correlation heat map of root-related indicators. **Significant at *p < 0.01*; *significant at *p < 0.05*.The same below.

Analyses of Pearson’s correlation coefficients by correlation heat map showed that the AMF colonization rate had a significant positive correlation with the root-related indices, such as the root DHA activity, total root length, average root diameter, root volume, and root surface area (**Figure 7**). Root biomass was also significantly positively correlated with the total root length and root volume. Root DHA activity was significantly positively correlated with the total length, projected area, average diameter, and volume of roots.

**Figure 8:**
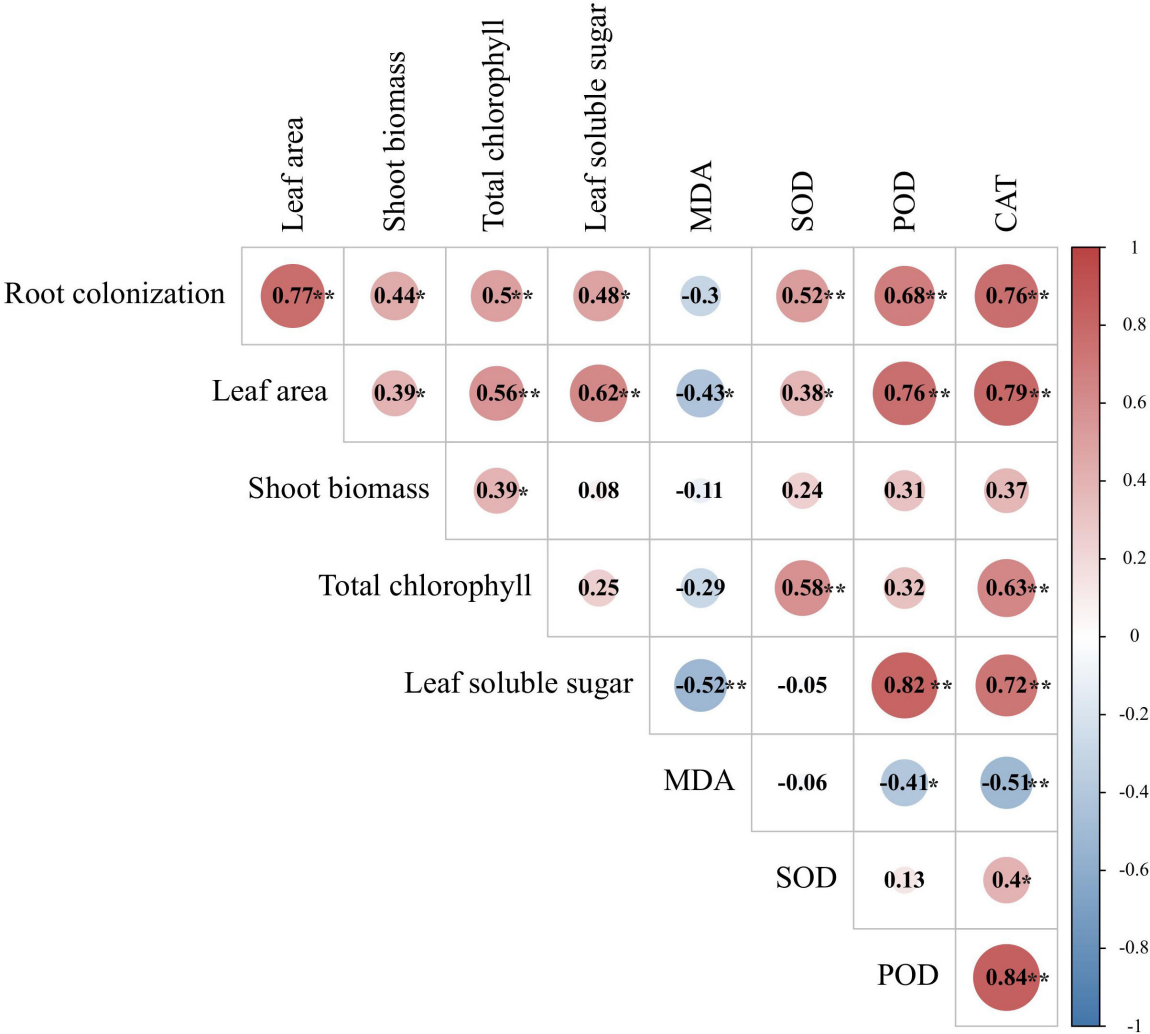
Correlation heat map between AMFs colonization and leaf-related indicators. **Significant at p < 0.01; *significant at p < 0.05.

The AMF colonization rate was also positively correlated with leaf-related indices, such as leaf surface area, total chlorophyll, leaf SOD, POD, and CAT activity, shoot biomass, and leaf soluble sugar content (**Figure 8**). Similarly, the leaf area exhibited significant positive correlations with the soluble leaf sugar content, leaf SOD, POD and CAT activity, shoot biomass, and total chlorophyll, while a significant negative correlation was observed between leaf area and MDA content. The total chlorophyll content was significantly positively correlated with leaf SOD and CAT activities. The soluble leaf sugar content was significantly positively correlated with leaf POD and CAT activity, while it was negatively correlated with leaf MDA content.

Collectively, the AMF colonization rate was significantly positively correlated with most of plant growth and physiobiochemical characteristics and could be one of the key factors affecting the growth status of tea cutting seedings.

## 4 Discussion

Both PAs and SLs can regulate the symbiosis between AMF and plants in various ways (Wu et al., 2012; Mitra et al., 2021a). This point of view was indirectly confirmed in our study. This study showed that regardless of AMF inoculation, the AMF structure was observed in the rhizosphere of Fuding-Dabaicha in all treatment groups, while the RI or FM inoculations promoted AMF colonization compared to the corresponding control treatments (Cao et al., 2021b). This was because in the field, although the seedling bed was treated with 5% formaldehyde for disinfection over 24 h before the test, a small quantity of AMF still survived. However, the effect of the surviving AMF was weaker than that of the additional AMF inocula, since the spore density of the inocula was quite higher than that in the treated seedling soil bed. The soil survived AMF could not meet the crop demands, and therefore, additional AMF inoculations are required to improve the mycorrhizal benefits to plants.

AMF-hormone interactions had a significant effect on the mycorrhizal colonization of the tea rhizosphere, which was consistent with the results of Kountche et al. (2018). SLs can induce AMF spore germination, mycelium elongation, and branching, and play a key role in the communication between plants and fungi (Mostofa et al., 2018). However, in this study, SL spraying significantly promoted the AMF colonization of rhizospheres in both the non-inoculated and RI-inoculated treatment groups, but did not promote the root colonization of FM, thus, indicating that the effect of hormones could be unique to specific AMF strains. In addition, exogenous PA treatment had no significant effect on the AMFs colonizing Fuding-Dabaicha roots, which was inconsistent with the results of Wu and Zou (2010) in citrus. Those findings could be attributed to differences in test materials. Mycorrhizal regulation by PAs has also been shown to depend on the types of polyamines and roots (Wu and Zou, 2010; Wu et al., 2012).

In this study, RI or FM inoculation, and the application of SL and PA, improved the quality and growth status of tea cutting seedlings to differing degrees. Once AMF achieve a symbiotic relationship with host plants, they expand the roots of hosts through mycelia, promoting nutrient and water absorption, stress resistance, and growth of the host plant (Wang et al., 2016; Chandrasekaran et al., 2021). SLs can promote seed germination, in shaping root architecture (Ruyter-Spira et al., 2011), regulate plant branching, and improve plant biomass (Kapulnik et al., 2011; Sharifi and Bidabadi, 2020). Similarly, PAs, as common low-molecular-weight biostimulation agents, promote plant growth, development, and defense under stress conditions (Wu et al., 2012; Sharma et al., 2021). In this study, PA spraying with RI or FM inocula had no significant impacts on the growth of tea cutting seedlings, while the interaction between RI and SL improved tea cutting seedling growth. Notably, SL induces spore germination and mycelium branching in AMFs, facilitating its symbiosis with the host plant (Mitra et al., 2021a). Hormone treatments can promote the benefits of AMF to tea plants by improving plant growth to different degrees. However, the degree of the effect varies based on the AMF species and hormones. Our results suggest that +RI + SL treatment may have a strong potential for the development of tea cuttings seedings and improving asexual propagation.

Adventitious root formation is a prerequisite for successful cutting propagation (Kharal and Shrestha, 2020), and a good root system architecture could provides sufficient nutrients to tea cutting seedings (Campos et al., 2021). In this study, the single inoculation of RI or FM significantly increased the projected root area, and the average diameter and volume of tea roots. In addition, spraying SL with the RI inocula further promoted the positive effect, because SL promoted the symbiotic relationship with the host plant (Mitra et al., 2021a). The SL synthesized in roots can then be transported to the soil over a short distance, so it could further promote mycorrhizal colonization (Shen et al., 2022). The root architecture was consistent with the growth quality of tea cuttings among all treatments. Correlation analyses also showed significantly positive correlations between AMF root colonization and root morphological traits and biomass(*P < 0.05*), thus, indicating that the SL-RI interaction promoted the growth of tea cutting seedlings related to the AMF colonization.

The root activity reflects the metabolic capacity of plant roots (Liu et al., 2009; Huang et al., 2015). AMF or SL treatments alone did not show a significant effect on root DHA activities (an indicator of root activity), while PA treatments alone elevated DHA activity. Notably, PAs are an important regulator of root growth and function (Sharma et al., 2021). The combination of SL or PA with RI inoculation significantly improved root DHA activity, along with providing better effects under the +RI + SL combined treatment. The DHA activity was significantly positively correlated with the total root length, projected area, and average diameter and volume, suggesting that SL conferred good root activity in mycorrhizal plants.

Plant growth is driven by photosynthesis (Moustakas et al., 2020). In this study, AMF inoculation increased the Pn and Cond of tea cutting seedlings, which was consistent with the results of Wu and Zou (2010) in citrus seedlings. Moreover, we found that the individual SL or PA treatments alone failed to change the photosynthetic activity of tea cutting seedlings, as the photosynthetic activity in combination with AMF treatments promoted almost the same change to the varying degrees. Notably, the +RI + SL treatment group showed the highest increase in the photosynthetic capacity of tea cutting seedlings. The increased photosynthetic capacity accelerates the accumulation of plant carbohydrates, promotes the growth of tea cutting seedlings, and increases the supply of organic carbon to AMFs, promoting mycorrhizal symbiosis with tea plants (Kountche et al., 2018). In addition, mycorrhiza-induction enhances root structure and plant hormones promote the absorption of soil nutrients, which increases photosynthesis in tea seedlings (Mitra et al., 2021b).

Root activity showed a significant positive correlation with the photosynthetic rate (Wei et al., 2004). Strong root activity also delays the senescence of above-ground plant parts and promotes the synthesis and partitioning of photosynthates (Wang et al., 2013). Therefore, the +RI + SL treatment group had higher chlorophyll and soluble sugar contents in leaves. Additionally, SL or PA alone significantly increased the total chlorophyll content, but reduced the soluble sugar content in tea leaves. In general, leaf-produced carbohydrates are first used for the leaf and then transferred to other parts of the plant (Zhang et al., 2020b). Thus, spraying SL or PA may have promoted the leaf carbohydrate distribution to the roots, increasing root development and thereby reducing leaf soluble sugar content.

The morphological and physiological adaptation of the root system are important factors for plants to access soil resources. Cutting seedings may experience poor nutrient absorption and poor stress tolerance, which will lead to the substantial production of ROS and affect photosynthesis because of the shallow root system and weak nutrient absorption (Mutui et al., 2015; Yang et al., 2016). AMF could improve the stress resistance of host plants by promoting antioxidase activities; reducing the MDA content is also well known (Yan et al., 2020). In this study, single RI inoculation noticeably promoted POD and CAT activities, while single FM inoculation promoted CAT activities and reduced MDA content. SL treatment alone promoted SOD and CAT activities, which was consistent with the findings of Sharifi and Bidabadi (2020). The synthetic exogenous SLs are mainly SL analogs (e.g., germination releaser, GR). GR24 is the most active and commonly used synthetic SL (Jiang et al., 2013) and its exogenous application has been shown to improve cell viability, photosynthesis, and antioxidant enzyme activities in tomato and rape seedlings (Lu et al., 2019; Zhang et al., 2020a). In this study, the foliar spray containing GR24 significantly improved the antioxidant enzyme activity and effectively alleviated oxidative damage in salvia. PA alone showed no significant effects on MDA content or antioxidase activity. However, PA treatment could maintain the cell pH and ionic homeostasis to ensure the maintenance of cell health (Zhang et al., 2020c). In addition, SL in combination with AMF further promoted the SOD, POD, and CAT activities of tea, thereby improving their survival rate and growth performance.

In addition, Pearson’s correlation analyses showed that the degree of root mycorrhizal colonization was significantly positively correlated with most plant growth and physiobiochemical characteristics parameters of tea cutting seedings, such as significantly positively correlated with root-related indicators (eg. root DHA activity, total root length, average root diameter, root volume) and leaf-related indicators(eg. leaf surface area, total chlorophyll index, and leaf SOD, POD, and CAT activity), so mycorrhizal colonization could be considered as one of the key factors reflecting the growth status of tea cutting seedings.

SLs, as host-derived precolonization signals (Akiyama et al., 2005), can stimulate the hyphal branching of AMF, and consequently promote symbiotic interactions between AMF and plants (Banasiak et al., 2020). Furthermore, during the symbiosis phase, SLs in root secretions enhance AMF spore germination, metabolic activity, and mycelium branching, thus, improving the interactions between AMFs and host plants (López-Ráez et al., 2017). The improved growth performance observed in +RI + SL treated tea cutting seedings can be attributed to the good symbiotic relationship between AMF and tea cutting seedings as the mycorrhizal colonization observed following this treatment was the highest compared to the corresponding AMF treatment alone. This good performance required to select a combination of specific AMF and specific plant hormone, for the mycorrhizal colonization in other AMF and hormone treatment(eg. +RI + PA, FM + SL) were not always higher or significantly increasing compared to corresponding AMF alone treatment. This could potentially be specific combination being case specific. For AMF-hormone interactions, especially between specific AMFs and the specific plant hormones, in our study were RI and SL, their effects on improving the growth performance many well by SL promoting the RI colonization to from good symbiosis with tea cutting seedings, then take this to improve their root architecture and photosynthetic characteristics, coupled with great antioxidant defense systems. The mechanisms needed to further study.

In conclusion, although the apllication of individual AMF or plant hormones had a certain good effects on most growth and physiobiochemical characteristics parameters of tea cutting seedings, the additive effect was from specific combined of AMF and plant hormone, in our study was the combined of SL and RI. These results highlight the possibility for combined of AMF and plant hormone to improve the asexual reproduction of tea plants *via* cuttings, and our findings provide a provided a practical and feasible strategy for further research to improve the asexual reproduction of tea plants *via* cuttings.

## Data availability statement

The original contributions presented in the study are included in the article/supplementary material. Further inquiries can be directed to the corresponding author.

## Author contributions

XG designed the experiment. YL and CL wrote the manuscript and revised the manuscript. CG and YZ prepared the materials for the experiment. CM and XD analyzed the data. All authors contributed to the article and approved the submitted version.
